# Simultaneous Genotyping of the rs4762 and rs699 Polymorphisms in Angiotensinogen Gene and Correlation with Iranian CAD Patients with Novel Hexa-primer ARMS-PCR

**Published:** 2017-06

**Authors:** Mehri KHATAMI, Mohammad Mehdi HEIDARI, Mehdi HADADZADEH, Barbara SCHEIBER-MOJDEHKAR, Morteza BITARAF SANI, Massoud HOUSHMAND

**Affiliations:** 1. Dept. of Biology, Faculty of Sciences, Yazd University, Yazd, Iran; 2. Dept. of Cardiac Surgery, Afshar Hospital, Shahid Sadoughi University of Medical Sciences, Yazd, Iran; 3. Dept. of Medical Chemistry, Medical University of Vienna, Vienna, Austria; 4. Dept. of Animal Science, University of Applied Science and Technology, Yazd, Iran; 5. Dept. of Medical Genetic, National Institute for Genetic Engineering and Biotechnology (NIGEB), Tehran, Iran

**Keywords:** Angiotensinogen gene, Coronary artery disease, rs4762, rs699, H-ARMS-PCR

## Abstract

**Background:**

A significant role of Renin-angiotensin system (RAS) genetic variants in the pathogenesis of essential hypertension and cardiovascular diseases has been proved. This study aimed to develop a new, fast and cheap method for the simultaneous detection of two missense single nucleotide polymorphisms (T207M or rs4762 and M268T orrs699) of angiotensinogen (*AGT)* in single-step Multiplex Hexa-Primer Amplification Refractory Mutation System - polymerase chain reaction (H-ARMS-PCR).

**Methods:**

In this case-control study, 148 patients with coronary artery disease (CAD) and 135 controls were included. The patients were referred to cardiac centers in Afshar Hospital (Yazd, Iran) from 2012 to 2015. Two sets of inner primer (for each SNP) and one set outer primer pairs were designed for genotyping of rs4762 and rs699 in single tube H-ARMS-PCR. Direct sequencing of all samples was also performed to assess the accuracy of this method. DNA sequencing method validated the results of single tube H-ARMS-PCR.

**Results:**

We found full accordance for genotype adscription by sequencing method. The frequency of the *AGT* T521 and C702 alleles was significantly higher in CAD patients than in the control group (OR: 0.551, 95% CI: 0.359–0.846, *P*=0.008 and OR: 0.629, 95% CI: 0.422–0.936, *P*=0.028, respectively).

**Conclusion:**

This is the first work describing a rapid, low-cost, high-throughput simultaneous detection of rs4762 and rs699 polymorphisms in *AGT* gene, used in large clinical studies.

## Introduction

Several studies have established the evidence of a genetic linkage between the angiotensinogen gene (AGT) and essential hypertension (1, 2). Angiotensinogen (AGT) is synthesized mainly by the liver and released into the circulation. More than 20 molecular variants have been identified in the *AGT* gene (3, 4). Polymorphisms of particular interest are a C-to-T transition at nucleotide 521 (C521T) that causes the change of threonine 207 to methionine (T207M or rs4762) and a T to C base substitution at nucleotide 702 (T702C) that causes the change of methionine 268 to threonine (M268T or rs699). Both polymorphisms are located in exon 2 on chromosome 1 found to be strongly associated with essential hypertension and the pathogenesis of coronary disease (5–7).

Different methods have been used to detect polymorphisms of AGT; among them are PCR amplification followed by restriction endonuclease digestion (8, 9), PCR-Single-strand conformation polymorphism (PCR-SSCP) (10, 11), real-time PCR (12, 13) and Denaturing Gradient Gel Electrophoresis (DGGE) (14) further contributed to the discrepancy in the results. These techniques are relatively slow and very expensive in comparison to Hexa-primer-amplification refractory mutation system-PCR (H-ARMS-PCR). With this method, the genotyping could be done using only a thermocycler machine at the least time.

In conventional ARMS PCR, the amplification of normal and mutant allele is done in two separate reactions, but in Multiplex ARMS-PCR, we can amplify both normal and mutant allele with a control fragment in a single reaction. The H-ARMS-PCR is performed by two non-allele-specific outer primers and four allele-specific inner primers in opposite orientation to each other. The outer primers amplify a large fragment of the target gene contains variant nucleotide as a control fragment and smaller allele-specific amplicons with different sizes that can easily be discriminated on gel electrophoresis either as homozygous or heterozygous. A deliberate mismatch at position −2 or −3 from the 3′ terminal end of the inner primers can improve allele specificity (15).

Since rs4762 (T207M) and rs699 (M268T) polymorphisms are located in exon 2 of *AGT* gene and the distance between them is 182 bp, we developed a new H-ARMS-PCR for simultaneous detection of these SNPs in single tube.

## Materials and Methods

### Subjects and DNA isolation

In this case-control study, we recruited 148 CAD patient candidates for coronary artery bypass graft (CABG) surgery selected from 282 cases who referred to cardiac centers in Afshar Hospital (Yazd, Iran) due to symptoms of myocardial infarction from 2012 to 2015. Patients had major lesions (>50% narrowing of luminal diameter) in one, two, or three vessels (left anterior descending (LAD), left circumflex artery (LCX), and right coronary artery (RCA)) that were candidate for surgery. CAD patients were identified according to the coronary angiography guidelines (16).

When up to 50% blockage was observed in the major epicardial coronaries and their branches associated with stenotic lesions, coronary arterial disease was considered present. The control group consisted of 135 unrelated healthy age, sex and ethnicity matched individuals with normal or near normal angiography reports (no lesion greater than 30%) (Table 1).

**Table 1:** The summary of the clinical characteristics of coronary atherosclerosis patients and controls

**Variable**	**Patients (n= 148)**	**Controls (n= 135)**
Male gender (%)	73	69
Age (yr)	52.6±7.2	51.9± 6.7
Smokers (%)	26	22
Body mass index (kg/m^2^) ^*^	26.5±2.6	25.3±2.1
Cholesterol, mg/dl ^*^	208.6±53.4	182.3±46.4
LDL-C, mg/dl	125.4±44.8	112.9±43.9
HDL-C, mg/dl	42.8±8.9	48.7±12.5
TGs, mg/dl ^*^	201.2±108.2	149.3±92.5
Systolic blood pressure (mmHg) ^*^	153.2±0.6	116.9±0.3
Diastolic blood pressure (mmHg)^*^	97.5±0.4	78.3±0.4

* *P-*value < 0.5

Yazd University Human Research Ethics Committee approved the study, in accordance with the revised declaration of Helsinki and all study participants gave their informed consents for the genetic analysis. Genomic DNA was extracted from peripheral blood samples using a salting out method.

### Primer Design

Two common missense SNPs (T207M or rs4762and M268T or rs699) of *AGT* gene were selected (https://www.ncbi.nlm.nih.gov/snp/). The H-ARMS-PCR method for detection of two *AGT* polymorphisms was performed according to the tetra-primer PCR procedure (17). Six primers were designed by the online website Primer 1:http://primer1.soton.ac.uk/primer1.html (Table 2). The specificity of the primers was checked by ‘BLAST’ program at http://www.ncbi.nlm.nih.gov/blast. To enhance the specificity of inner primers, a destabilizing mismatch was incorporated at the third nucleotide from the 3′-terminus of each primer.

**Table 2:** PCR primer sequences

**AGT gene**		**Primer sequence**	**Amplicon size**
(SNP ID: rs4762)	Forward inner primer C allele	5′-GGCCCAGCTGCTGCTGTCAAC-3′	672 bp
	Reverse inner primer T allele	5′-GCTGTGAACACGCCCACCAACA-3′	245 bp
(SNP ID: rs699)	Forward inner primer T allele	5′-GGAAGACTGGCTGCTCCCTTAC-3′	490 bp
	Reverse inner primer C allele	5′-GTGCTGTCCACACTGGCTCACA-3′	428 bp
Control Fragment	Forward outer primer	5′-GTCCTCTCCCCAACGGCTGTCT-3′	875 bp
	Reverse outer primer	5′-AACCTGACCCTTCTGAGTGTAG-3′	

### H-ARMS-PCR Analysis

PCR was performed in a total volume of 25 μL containing 50 ng of template DNA, 5 pmol of each outer primers, 10 pmol of each inner primers, 1X Multiplex PCR Master Mix (Yekta Tajhiz Azma Co., Tehran, Iran).

PCR amplification (touchdown) was carried out at 95 °C for 2 min, followed by denaturation at 95 °C for 20 sec, first annealing at 70 °C to 61 °C (10 cycles). In the remaining cycles (25 cycles) annealing was carried out at 60 °C for 1 min and extension at 72 °C for 1 min, followed by a final extension for 5 min. Non-denaturation polyacrylamide gel electrophoresis (6%) and silver staining was used for separation and detection of PCR products.

The rs4762 (C521T) and rs699 (T702C) polymorphisms are located in exon 2 *AGT* gene with 182 bp distance. Two primers, F1 and R1 were designed to amplify an 875 bp band, which served as control fragment for the success of amplification of two polymorphisms. Four specific primers, F2 and R2 for rs4762 and F3 and R3 for rs699, with complementary 3′-termination to corresponding polymorphisms, were introduced (Table 2).

**Fig. 1:**
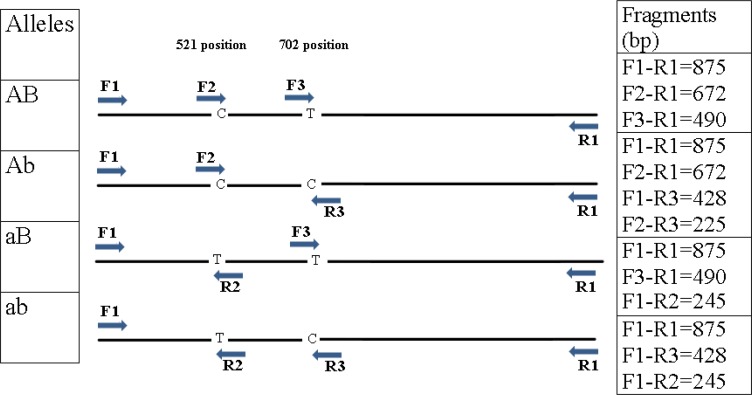
Schematic illustration of primer interactions for Multiplex H-ARMS-PCR assay F1 and R1 are outer primers acting as control primers, and F2 and R2 are inner primers for 521 nucleotide substitution (rs4762, C521T) and F3 and R3 are inner primers for 702 nucleotide substitution (rs699, T702C)

Here, we designate *AGT* gene with C and T nucleotide in position 521(C521T) as A and an allele respectively, and T and C nucleotide in position 702 (T702C) as B and b allele respectively. Therefore, four allele compositions are recognized for *AGT* gene including AB, Ab, aB and ab (schematic illustration in Fig. 1) and generated nine possible genotypes – AABB, AaBB, aaBB, aaBb, AABb, Aabb, AaBb, Aabb and aabb. The results were validated with direct DNA sequencing by using ABI 3700 capillary sequencer in 10% of samples.

### Statistical Analysis

Unpaired Student’s *t*-tests were used for comparison of distributions of continuous variables (as mean ± SD) in groups. The *AGT* gene SNPs was first assessed in three genotype categories (wild-type, heterozygote, and homozygote SNPs) and then grouped into two categories with heterozygotes and homozygote variants combined because of the dominant, the recessive, overdominant and log-additive models of inheritance observed for these polymorphisms. The association between two groups were examined by Chi-square goodness-of-fit test. Multiple logistic regression models (co-dominant, dominant and recessive) were employed to analyze the genetic data. Values of *P*<0.05 were regarded as statistically significant. The SPSS software (IBM SPSS 22, SPSS Inc., Chicago, IL, USA) was used for statistical analysis.

## Results

The identification of each genotype (and Cis or Trans-state) was performed by comparing to the expected fragment lengths (Table 3).

**Table 3:** Amplification patterns observed and those expected according to AGT polymorphism genotypes. A and a alleles are C and T nucleotides in position 521 (rs4762, C521T), respectively; B and b alleles are T and C nucleotides in position 702 (rs699, T702C), respectively; Cis is order of nucleotides as AB or ab in chromosome and Trans is order of nucleotides as Ab or aB in chromosome.

**Genotype**	**AABB**	**AABb**	**AAbb**	**AaBB**	**AaBb**		**Aabb**	**aaBB**	**aaBb**	**aabb**
Cis or Trans	AB/AB	AB/Ab	Ab/Ab	AB/aB	AB/ab	Ab/aB	Ab/ab	aB/aB	aB/ab	ab/ab
CommonAmplic(bp)					875					
	672	672	672	672	672	672	672			
	490	490		490	490	490		490	490	
Amplicon (bp)		428	428		428	428	428		428	428
				245	245	245	245	245	245	245
		225	225			225	225			

**Fig. 2:**
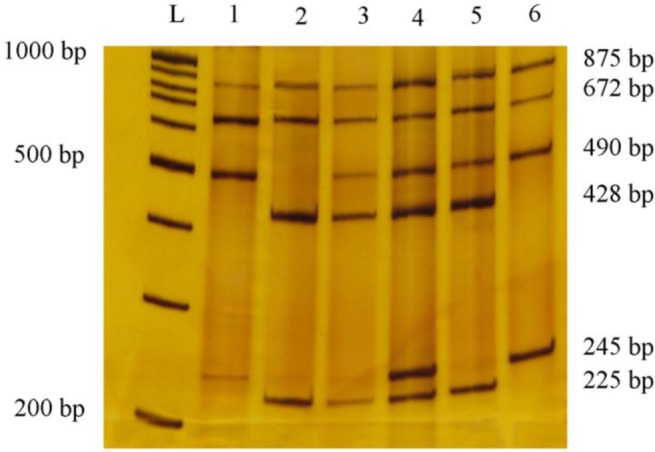
Results of H-ARMS-PCR of *AGT* polymorphisms (rs4762 and rs699) Lanes 1 and 6: 521CT heterozygote and 702TT homozygote (AaBB genotype), Lane 2: 521CC homozygote and 702TT homozygote (AAbb genotype), lanes 3 and 5: 521CC homozygote and 702TC heterozygote (AABb genotype) and Lane 4: 521CT homozygote and 702TC homozygote (AaBb-Ab/aB genotype)

A and a alleles are C and T nucleotides in position 521 (rs4762, C521T), respectively; B and b alleles are T and C nucleotides in position 702 (rs699, T702C), respectively; Cis is order of nucleotides as AB or ab in chromosome and Trans is order of nucleotides as Ab or aB in chromosome. On the base of the nucleotide substitution at positions 521 and 702 in the *ATG* gene, one pair outer primer and two pairs of inner primers can amplify related fragments. AB alleles introduced 875, 672 and 490 bp, Ab alleles 875, 672, 428 and 225 bp, aB alleles 875, 490 and 254 bp and ab alleles 875, 428 and 254 bp (Fig. 2). Ten percent of samples analyzed with H-ARMS-PCR were reevaluated by direct sequencing analysis. We obtained 100% accordance with both methods for genotype adscription.

Coronary angiography revealed 148 patients (CAD^+^ group) with one-vessel (LAD) (n= 35), two-vessels (LCX) (n= 59), or three-vessels (RCA) (n= 54) that were candidate for CABA (Coronary Artery Bypass Graft) and 135 patients (CAD^−^ group) with no angiographically identified narrowing. Mean age (mean ± SD) was 53.9 ± 6.8 and 52.6 ± 6.9 yr for patients and controls, respectively.

Genotype distributions and allelic frequencies of *AGT* polymorphisms among CAD patients and controls are shown in Table 4. The frequency of the *AGT* T521 and C702 alleles was significantly higher in the CAD patients than in control group (OR: 0.551, 95%CI: 0.359–0.846, *P*=0.008 and OR: 0.629, 95% CI: 0.422–0.936, *P*=0.028, respectively).

**Table 4:** Genotype and allele frequencies of AGT variants in patients and controls

***AGT (rs4762)***	**Patients (n=148)**	**Controls (n=135)**	**OR (95% CI)**	***P-value***
Codominant model				
CC	86 (58.1)	98 (72.6)	1	
CT	53(35.8)	4 (25.2)	0.591 (0.348–1.005)	0.52
TT	9 (6.1)	3 (2.2)	0.258 (0.067–0.996)	0.49
Dominant model				
CC	86 (58.1)	98 (72.6)		
CT+ TT	62 (41.9)	37 (27.4)	1.91 (1.16–3.15)	0.01
Recessive model				
CC+CT	139 (93.9)	132 (97.8)		
TT	9 (6.1)	3 (2.2)	2.85 (0.76–1075)	0.09
Over-dominant model				
CC+ TT	95 (64.2%)	101 (74.8)		
CT	53 (35.8%)	34 (25.2)	1.66 (0.99–2.77)	0.05
Allele frequency				
C	225 (76)	230 (85.2)		
T	71 (24)	40 (14.8)	0.551 (0.359–0.846)	0.008
***AGT* (rs699)**				
Co-dominant model				
TT	83 (56.1)	94 (69.6)	1	
TC	50 (33.8)	31 (23)	0.567 (0.327–0.980)	0.042
CC	15 (10.1)	10 (7.4)	0.525 (0.222–1.244)	0.143
Dominant model				
TT	83 (56.1)	94 (69.6)		
TC+CC	65 (43.9)	41 (30.4)	1.80 (1.10–2.93)	0.01
Recessive model				
TT+TC	133 (89.9)	125 (92.6)		
CC	15 (10.1)	10 (7.4)	1.41 (0.61–3.25)	0.14
Over-dominant model				
TT+CC	98 (66.2)	104 (77)		
TC	50 (33.8)	31 (23)	1.01 (1.01–2.90)	0.04
Allele frequency				
T	216 (73)	219 (81.1)		
C	80 (27)	51 (18.9)	0.629 (0.422–0.936)	0.028

The Multiple logistic regression analysis showed a significant association of *AGT* polymorphisms and CAD according to dominant model for rs4762 and rs699 (OR: 1.91, 95%CI: 1.16–3.15; *P*=0.01 and OR: 1.80, 95%CI: 1.10–2.93; *P*=0.01, respectively) (Table 3).

Table 5 shows the results of the haplotype analysis. We found that AABb and aaBb haplotypes might significantly increase risk of CAD (*P*=0.029, OR: 0.460, 95%: 0.229–0.925, and OR: 3.146, 95% CI: 3.146–3.146, respectively).

**Table 5:** Haplotype analysis of AGT polymorphisms in CAD patients and controls

**Genotype**	**Cis or Trans**	**Patients(n=148)**	**Controls(n=135)**	**OR (95% CI)**	***P*-value**
AABB	AB/AB	49	71	1	
AABb	AB/Ab	26	18	0.460 (0.229–0.925)	0.029
AAbb	Ab/Ab	11	9	0.531 (0.267–1.056)	0.071
AaBB	AB/aB	27	20	0.478 (0.190–1.204)	0.117
AaBb	AB/ab	13	9	0.565 (0.218–1.465)	0.240
	Ab/aB	9	4	0.307 (0.089–1.052)	0.060
Aabb	Ab/ab	4	1	0.173 (0.019–1.591)	0.121
aaBB	aB/aB	7	3	0.296 (0.073–1.200)	0.088
aaBb	aB/ab	2	0	3.146 (3.146–3.146)	0.000
aabb	ab/ab	0	0	-	-

OR, odds ratio; CI, Confidence interval.

## Discussion

The AGT variants are considered to be a risk factor in various diseases and to be significant in body fluid hemostasis. Haplotype analysis showed a significant association between *AGT* rs4762 and rs699 with hypertension among Caucasian and Taiwan Chinese populations (18–20). The standard PCR instruments are highly desirable for scientific studies of large numbers of patients and for diagnostic analyses, economical and fast assays. Because these polymorphisms are risk factors for much morbidity, including cardiovascular disease and hypertension, here, we developed a rapid, sensitive and one-step hexa-primer PCR-ARMS method for detection rs4762 and rs699 polymorphisms.

The most critical step for successful development of a new multiplex amplification refractory mutation system-PCR method is the primer design. In this study, the primers for amplification of *AGT* rs4762 and rs699 polymorphisms were designed using the online website Primer1: http://primer1.soton.ac.uk/primer1.html.

**Fig. 3:**
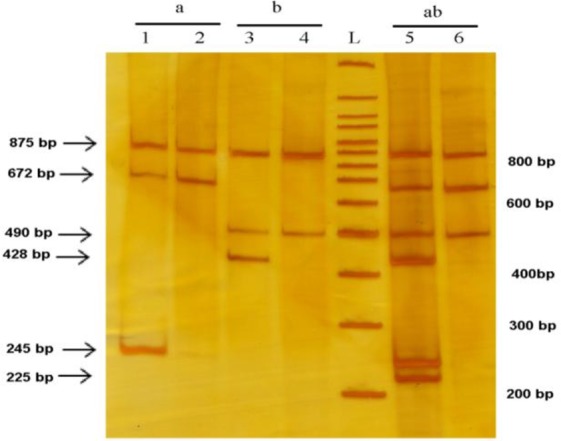
Determination of primer sensitivity and specificity in separate tetra-primer ARMS –PCR assay for detection rs4762 and rs699 polymorphisms Line an indicates T-ARMS-PCR assay for detection rs4762 polymorphism with F1, R1, F2 and R2 primers. Lane 1 is heterozygous state and lane 2 is homozygous state. Line b indicates T-ARMS-PCR assay for detection rs699 polymorphism with F1, R1, F3 and R3 primers. Lane 3 is heterozygous state and lane 4 is homozygous state. Lines ab indicate H-ARMS-PCR assay for simultaneous detection rs4762 and rs699 polymorphisms with F1, R1, F2, R2, F3 and R3 primers. Lane 5 is heterozygous state for each two SNPs and lane 6 is homozygous state for each two SNPs

The primer sensitivity, specificity, and the annealing temperature were tested using separate tetra-primer ARMS-PCR for each SNP. In one reaction, T-ARMS-PCR assay was performed with F1, R1, F2 and R2 primers for detection of rs4762 polymorphism (Fig. 3, Line a) and in the other reaction, the T-ARMS-PCR assay was performed with F1, R1, F3 and R3 primers for detection of rs699 polymorphism (Fig. 3, Line b).

**Fig. 4:**
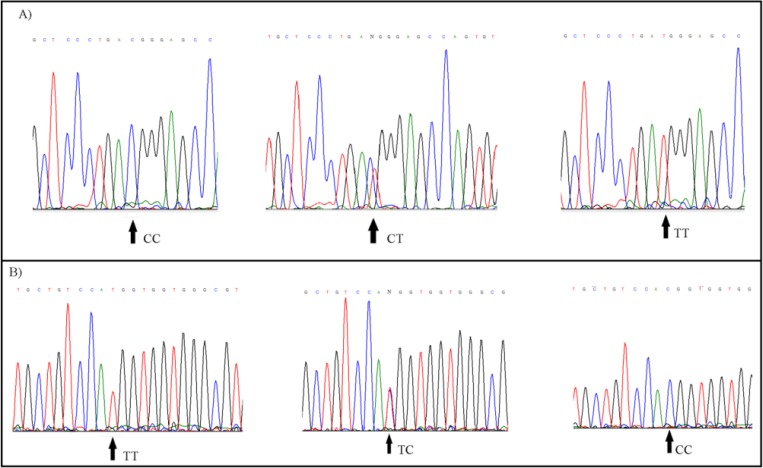
The results of sequencing were consistent with genotypes of the *AGT* rs4762 (A) and rs699 (B) variants determined by H-ARMS-PCR

In addition, we obtained 100% accordance between direct DNA sequencing and H-ARMS-PCR for genotype adscription (Fig. 4). This assay is a reproducible and stable single-tube reaction.

## Conclusion

We needed only a small amount of traditional PCR reagents and no special equipment. This procedure could be used by most clinical diagnostic laboratories. Additionally, this H-ARMS-PCR could be accomplished within 2.5 h following sample receipt, time-saving in the laboratory.

## Ethical considerations

Ethical issues (Including plagiarism, informed consent, misconduct, data fabrication and/or falsification, double publication and/or submission, redundancy, etc.) have been completely observed by the authors.
